# What is the burden of multimorbidity and the factors associated with its occurrence in elderly Brazilians?

**DOI:** 10.1590/0034-7167-2022-0809

**Published:** 2024-05-03

**Authors:** Danielle Samara Tavares de Oliveira-Figueiredo, Matteus Pio Gianotti Pereira Cruz Silva, Paula Yhasmym de Oliveira Feitosa, Bruna Cardoso Leite, Fabiana Lucena Rocha, Luciana Dantas Farias de Andrade

**Affiliations:** I Universidade Federal de Campina Grande. Cuité, Paraíba, Brazil; II Universidade Federal de Campina Grande. Cajazeiras, Paraíba, Brazil; III Universidade Federal da Paraíba. João Pessoa, Paraíba, Brazil

**Keywords:** Aged, Multimorbidity, Chronic Disease, Multiple Chronic Conditions, Cross-Sectional Studies, Anciano, Multimorbilidad, Enfermedad Crónica, Afecciones Crónicas Múltiples, Estudios Transversales, Idoso, Multimorbidade, Doença Crônica, Múltiplas Afecções Crônicas, Estudos Transversais

## Abstract

**Objective:**

To estimate the prevalence of multimorbidity in elderly people and its association with sociodemographic characteristics, lifestyle, and anthropometry.

**Methods:**

This was a cross-sectional study using data from the National Health Survey, 2019. A total of 22,728 elderly individuals from all 27 Brazilian states were randomly selected. Poisson regression models with robust variance were employed, and a significance level of 5% was adopted.

**Results:**

The prevalence of multimorbidity was 51.6% (95% CI: 50.4-52.7), with the highest estimates observed in the South and Southeast. Multimorbidity was associated with being female (aPR = 1.33; 95% CI: 1.27-1.39), being 80 years old or older (aPR = 1.12; 95% CI: 1.05-1.19), having low education (aPR = 1.16; 95% CI: 1.07-1.25), past cigarette use (aPR = 1.16; 95% CI: 1.11-1.21), insufficient physical activity (aPR = 1.13; 95% CI: 1.06-1.21), and screen use for 3 hours or more per day (aPR = 1.13; 95% CI: 1.08-1.18).

**Conclusion:**

Multimorbidity affects more than half of the elderly population in Brazil and is associated with social, demographic, and behavioral factors.

## RESUMO

**Objetivo:**

Estimar a prevalência de multimorbidade em pessoas idosas e sua associação com características sociodemográficas, estilo de vida e antropometria.

**Métodos:**

Estudo transversal, com dados da Pesquisa Nacional de Saúde, 2019. Foram selecionadas aleatoriamente 22.728 pessoas idosas dos 27 estados brasileiros. Empregaram-se modelos de regressão de Poisson com variância robusta e adotou-se um nível de significância de 5%.

**Resultados:**

A prevalência de multimorbidade foi de 51,6% (IC95%: 50,4-52,7), sendo as maiores estimativas observadas no Sul e Sudeste. A multimorbidade foi associada ao sexo feminino (RPa=1,33; IC95%: 1,27-1,39), ter 80 anos ou mais (RPa= 1,12; IC95%: 1,05-1,19), baixa escolaridade (RPa=1,16; IC95%:1,07-1,25), consumo de cigarro no passado (RPa=1,16; IC95%:1,11-1,21), prática insuficiente de atividade física (RPa= 1,13; IC95%:1,06-1,21) e uso de telas por 3 horas ou mais por dia (RPa=1,13; IC95%:1,08-1,18).

**Conclusão:**

A multimorbidade afeta mais da metade da população idosa do Brasil e está associada a fatores sociais, demográficos e comportamentais.

## RESUMEN

**Objetivo:**

Estimar la prevalencia de multimorbilidad en personas mayores y su asociación con características sociodemográficas, estilo de vida y antropometría.

**Métodos:**

Estudio transversal, con datos de la Encuesta Nacional de Salud, 2019. Se seleccionaron aleatoriamente 22.728 personas mayores de los 27 estados brasileños. Se emplearon modelos de regresión de Poisson con varianza robusta y se adoptó un nivel de significancia del 5%.

**Resultados:**

La prevalencia de multimorbilidad fue del 51,6% (IC95%: 50,4-52,7), siendo las mayores estimaciones observadas en el Sur y Sudeste. La multimorbilidad se asoció con el sexo femenino (RPa=1,33; IC95%: 1,27-1,39), tener 80 años o más (RPa= 1,12; IC95%: 1,05-1,19), baja escolaridad (RPa=1,16; IC95%:1,07-1,25), consumo de cigarrillo en el pasado (RPa=1,16; IC95%:1,11-1,21), práctica insuficiente de actividad física (RPa= 1,13; IC95%:1,06-1,21) y uso de pantallas por 3 horas o más al día (RPa=1,13; IC95%:1,08-1,18).

**Conclusión:**

La multimorbilidad afecta a más de la mitad de la población anciana de Brasil y está asociada a factores sociales, demográficos y conductuales.

## INTRODUCTION

Multimorbidity refers to the simultaneous presence of two or more chronic conditions in an individual. This can include non-communicable chronic diseases (NCDs), long-term mental health conditions, and chronic infectious diseases^(1)^. The concept of multimorbidity differs from comorbidity, as the latter implies the combined effect of additional conditions in relation to the individual’s initial health condition^(2)^.

The rapid aging of the population, combined with unhealthy lifestyles and a high prevalence of NCDs, makes multimorbidity a challenge for health systems worldwide and a priority on research agendas^(1-3)^. This condition can increase the likelihood of disabilities and reduce the quality of life, leading to a higher risk of premature death^(3-4)^. Additionally, compared to peers with no or just one chronic disease, individuals with multimorbidity are more likely to experience prolonged hospitalizations and are associated with depression and the intake of multiple medications (polypharmacy), which increases healthcare costs^(5)^.

The prevalence of multimorbidity is rising, particularly among the elderly population. Much of the research on this issue focuses on high-income countries^(1)^. The global prevalence of this outcome in adults is 37.2%, with South America having the highest frequency^(6)^. Among the elderly, this condition affects 51% of the global population^(6)^. In Brazil, some population-based studies have been conducted with the general population, including adults and the elderly, with estimates of multimorbidity prevalence varying between 10.2% and 24.2%, depending on the number of health conditions used to measure this outcome^(4,7)^.

Prevalence studies of multimorbidity specifically in the elderly, with nationally distributed samples, are still scarce^(4,8)^. A study using cross-sectional data from 2008 found a prevalence of 81.3% for the occurrence of two or more morbidities and 64.0% for three or more in the elderly (4). Other studies with this age group have been conducted with samples limited to a single municipality (8) or a region of the country^(9)^.

Moreover, much of the previous research uses a restricted definition of multimorbidity, defining it only as the simultaneity of two or more NCDs^(9-10)^, without considering recent recommendations to standardize the definition of multimorbidity, which should include not just NCDs but also other chronic conditions like chronic mental diseases and chronic infectious diseases^(1)^. The divergence in the definition of multimorbidity in studies prompts the need for research that considers the new definition and the regional and local characteristics, as prevalences can vary based on geographic region and age group studied.

The high burden of multimorbidity in South America suggests the need for studies in Brazil focusing on the elderly population, as this group is more frequently affected by two or more chronic health conditions^(6)^. Therefore, there is a need for national data to support the gathering of information, monitoring, and evaluation of the multimorbidity profile in this population, in order to facilitate priority interventions that reduce premature mortality due to chronic conditions and multimorbidity.

These gaps, combined with the progressive aging of the population, make the prevention of chronic conditions, the maintenance of quality of life, and functionality crucial for the well-being and health of the elderly. Thus, studies with a representative sample of the Brazilian elderly population are useful to support collective health actions and policies, aimed at working with priority and more vulnerable groups, and to enhance the understanding of modifiable individual characteristics that can be targets for political and healthcare interventions by health professionals and nurses.

## OBJECTIVE

To estimate the prevalence of multimorbidity in elderly individuals and its association with sociodemographic characteristics, lifestyle, and anthropometry.

## METHODS

### Ethical Aspects

This research was conducted using secondary data available in the public domain on the website of the Brazilian Institute of Geography and Statistics (IBGE), thus not requiring approval from an Ethics Committee. Ethical review and approval were waived for this study, as it involved secondary data. Informed consent had already been obtained by the National Health Survey, which originated these data.

### Design, Period, and Location of the Study

This is a cross-sectional, analytical study using data from the 2019 National Health Survey (PNS), guided by the Strengthening the Reporting of Observational Studies in Epidemiology (STROBE) tool. The PNS is a health survey based on household interviews, representative of the Brazilian population, conducted in all states of Brazil, in both urban and rural areas, between 2019 and 2020^(11)^.

### Population or Sample, Inclusion and Exclusion Criteria

The PNS sampling plan was a three-stage clustered sample. Census sectors formed the primary sampling units (PSUs), totaling 8,036^(11)^. Within each PSU, a fixed number of private permanent households were randomly selected (15 households/PSU or 18 households/PSU, depending on the Brazilian state)^(11)^. A total of 108,457 households were selected throughout Brazil, with 94,114 household interviews conducted^(11)^. In each household, one resident aged 15 years or older was randomly selected to respond to a specific questionnaire^(11)^. A total of 90,846 individual interviews with the selected resident were conducted^(11)^.

Excluded from the PNS were households located in census sectors with small populations (such as indigenous areas, barracks, lodgings, camps, boats, penitentiaries, penal colonies, military bases, prisons, jails, long-term care institutions for the elderly, integrated care networks for children and adolescents, convents, hospitals, etc.)^(11)^.

The population used in this study comprised 90,846 individuals who responded to the individual interview in the third stage of the PNS selection. As an eligibility criterion for this study, elderly individuals (aged 60 years or older) from all Brazilian states who responded to the interviews were included in the sample, excluding responses given by third parties who were not the elderly person themselves. Additionally, adults and adolescents were excluded. Therefore, the sample used consisted of 22,728 elderly individuals from the community, selected by simple random sampling in all states of Brazil, participants in the third stage of selection of the PNS, 2019.

### Study Protocol

Data collection occurred between August 2019 and March 2020. The data collection instrument of the PNS is available at: https://www.pns.icict.fiocruz.br/. For details about the instrument, interviews, and data collection, refer to the PNS methodological publication^(11)^.

In this study, data from the PNS modules containing the general characteristics of the residents (module C), educational characteristics of people aged 5 years or older (module D), lifestyle (module P), and chronic diseases (module Q) were used. Data were collected by previously trained collection agents from the Brazilian Institute of Geography and Statistics (IBGE), with the assistance of handheld computers^(11)^.

To conduct the interview, the IBGE collection agent established contact with the head of the household or another resident, explaining the objectives, procedures, and the importance of participating in the research. All household members were listed using the microcomputer, identifying the authorized respondent to answer the household questionnaire and the questionnaire about data for all household members. Additionally, one resident aged 15 years or older was selected to respond to the individual interview using the random selection program of the handheld microcomputer^(11)^.

The dependent variable of this study was multimorbidity, defined as the simultaneous presence of two or more chronic health conditions in the same individual^(1,3)^. Chronic conditions were investigated through self-reports by elderly individuals about the medical diagnosis of the following conditions: diabetes, heart diseases (heart attack, angina, and congestive heart failure), systemic arterial hypertension, stroke, arthritis or rheumatism, cancer, emphysema/bronchitis or chronic obstructive pulmonary disease (COPD), chronic kidney failure, asthma or asthmatic bronchitis, chronic spinal problems, depression, and other mental illnesses (schizophrenia, bipolar disorder, psychosis, or Obsessive-Compulsive Disorder).

In the PNS, for each chronic condition of the elderly, the question asked was: Has a doctor ever diagnosed you with this condition? The presence of a chronic spinal problem was assessed by the following question: Do you have any chronic spinal problems, such as chronic back or neck pain, lower back pain, sciatic pain, vertebral or disc problems? For depression, the elderly person was asked if any doctor or mental health professional (such as a psychiatrist or psychologist) had ever diagnosed them with depression.

Multimorbidity was constructed from a score resulting from the summation of these chronic conditions. All variables of the chronic conditions were dichotomized into: 0 - does not report the diagnosis of a chronic condition; and 1 - has a self-reported diagnosis of the respective chronic condition. From this variable, the outcome variable of multimorbidity was dichotomized into: 0 - none or up to one chronic condition; and 1 - two or more chronic conditions.

The independent variables used were: Sex (coded as 0 - Male and 1 - Female); Age group (categorized into: 0 - 60 to 69 years, 1 - 70 to 79 years, and 2 - 80 years or older); Level of education (categorized by years of study: 0 - 12 years or more, 1 - 9 to 11 years, and 2 - 0 to 8 years of study); Self-reported skin color (categorized into: 0 - White, 1 - Black, 2 - Mixed, and 3 - Yellow or Indigenous); Marital status (0 - With partner and 1 - Without partner, including singles, divorced, and widowed); Region of residence (0 - South, 1 - Southeast, 2 - Central-West, 3 - North, and 4 - Northeast); Residential area (0 - Urban and 1 - Rural).

Socioeconomic classification was calculated from a score created using a scale from the Brazilian Association of Research Companies (ABEP)^(12)^, which uses questions about household assets (available in PNS module A) and the educational level of the resident. This classification stratifies socioeconomic levels into categories A to E, with class A being the highest socioeconomic level and E the lowest. In the research, socioeconomic class was categorized into 0 - A, 1 - B, and 2 - C, D, and E.

Lifestyle variables included: tobacco use (categorized into: 0 - Never Smoked, 1 - Currently Smokes, and 2 - Smoked in the Past) and alcohol consumption (categorized into: 0 - Never consumes, 1 - Light/Moderate consumption, and 2 - Heavy consumption). Consumption was considered heavy when there was an intake of five or more doses (for men) or four or more doses (for women) on a single occasion, at least once in the last month. This variable was constructed from questions in the PNS about the frequency and quantity of alcohol consumption^(13)^.

Physical activity practice was categorized into: 0 - Sufficient and 1 - Insufficient. Physical activity was defined as insufficient if it was less than 150 minutes/week of light or moderate activity, or less than 75 minutes of vigorous activity. Light or moderate activities included walking, weight training, water aerobics, dance, and general gymnastics; vigorous intensity activities included running, team sports, aerobic gymnastics, among others that increase heart rate beyond resting levels^(14)^.

Body Mass Index (BMI) was calculated by dividing weight (in kilograms) by the square of height (in meters). BMI was categorized into: 0 - Normal weight (≥18.5 kg/m^2^ and ≤24.9 kg/m^2^); 1 - Underweight (<18.5 kg/m^2^); and 2 - Overweight/Obesity (≥25.0 kg/m^2^), according to guidelines from the World Health Organization^(15)^. Screen time was defined as the average daily hours spent watching TV and using tablets, smartphones, and computers for leisure^(16)^, categorized into: 0 - Less than 3 hours/day and 1 - More than 3 hours/day.

### Analysis of Results and Statistics

Descriptive analyses of the exposures and the outcome were conducted, estimating the proportions of the outcome and their respective 95% Confidence Intervals (CI95%), stratified by sociodemographic characteristics. The Pearson Chi-square test was used in the bivariate stage to test associations between sociodemographic variables and multimorbidity.

Unadjusted association analyses between the independent variables and the outcome were performed using Poisson regression with robust variance. The prevalence ratio (PR) was used to quantify the relationship of the exposures with the outcome, with multimorbidity being a high-frequency event in the study population. The use of this robust estimator minimizes the risk of overestimation of the association, unlike the odds ratio (OR), which could also be employed^(17)^.

Variables with a p-value <0.20 in the bivariate analysis were included in the Poisson regression model with robust variance. Unadjusted and adjusted PRs were calculated for all variables included in the multiple model, which could also act as confounding variables, such as sex and age group.

The introduction of variables into the model started with the outcome, followed by the exposures of interest. Variables that remained associated with a significance level of less than 5% were considered associated with multimorbidity and included in the final model adjusted for all variables of the multiple model. The analyses were performed in Stata, version 17, using the Survey module, which allows for the use of sample weights for calibration of the complex sample design. For the presentation of prevalence maps of multimorbidity by Brazilian state, the QGis 3.26 program was used.

## RESULTS

This study involved 22,728 elderly individuals, representing a population estimate of 34,398,853 individuals. A predominance of females (55.5%; 95% CI: 54.5-56.5) was observed in the age group of 60 to 69 years (54.8%; 95% CI: 53.8-55.8), and among those with self-reported white skin color (51.3%; 95% CI: 50.2-52.4). The majority lived without a partner (56.7%; 95% CI: 55.7-57.7), had low educational levels, with zero to eight years of study (70.4%; 95% CI: 69.3-71.5), and were in the low socioeconomic level - classes C, D, and E (84.7%; 95% CI: 83.5-85.7), residing in urban areas (85.5%; 95% CI: 84.3-86.7) and in the Southeast region (47.3%; 95% CI: 46.3-48.3).

The prevalence of multimorbidity among the elderly in Brazil was 51.6% (95% CI: 50.4-52.8), being higher among older women (57.9%; 95% CI: 56.3-59.5), in the age group of 80 years or older (57.4%; 95% CI: 54.6-60.2), among those with low education - zero to eight years of study (53.6%; 95% CI: 52.4-54.8), with yellow skin color (55.0%; 95% CI: 46.4-63.6), among those living without a partner (53.6%; 95% CI: 52.2-55.0), belonging to lower social classes - C, D, and E (52.6%; 95% CI: 51.5-53.7), residing in urban areas (52.5%; 95% CI: 51.3-53.7), and in the South region of the country (54.1%; 95% CI: 51.9-56.3). Statistically significant differences were observed between sex (p=0.0001), age group (p<0.0001), marital status (p=0.0004), educational level (p<0.0001), social class (p=0.0023), residential area (p<0.0001), and region of residence (p<0.0001) in relation to the occurrence of multimorbidity (Table 1).

**Table 1:** Sociodemographic Characterization and Prevalence of Multimorbidity According to Sociodemographic Variables of the Elderly. Brazilian States, Brazil, 2019

Sociodemographic Variables	n	%^a^	95%CI^b^	Multimorbidity		
				% ^a^	95%CI ^b^	*p* value^c^
Total	22,728	100	-	51.6	50.4-52.7	-
Gender						
Male	10,193	44.5	43.5 - 45.5	43.2	41.6 - 44.8	<0.0001
Female	12,535	55.5	54.5 - 56.5	57.9	56.3 - 59.3	
Age Group						
60 to 69 years	12,555	54.8	53.8 - 55.9	48.1	46.5 - 49.5	<0.0001
70 to 79 years	7,157	31.1	30.2 - 32.0	55.6	53.6 - 57.4	
80 years or more	3,016	14.1	13.4 - 14.8	57.4	54.5 - 60.4	
Skin Color						
White	9,901	51.3	50.2 - 52.4	51.1	49.4 - 52.7	0.727
Black	2,455	10.2	9.62 - 10.8	52.3	48.8 - 55.7	
Brown	10,001	36.7	35.7 - 37.7	51.9	50.1 - 53.6	
Other^*^	369	1.8	1.6 - 2.1	55.0	46.3 - 63.3	
Marital Status						
With partner	9,946	43.3	42.3 - 44.3	49.7	48.0 - 51.3	0.0004
Without partner	12,782	56.7	55.7 - 57.7	53.6	52.1 - 55.1	
Education						
12 years or more	2,701	13.1	12.2 - 14.0	44.8	41.6 - 48.1	<0.0001
9 to 11 years	3,613	16.5	15.8 - 17.4	48.6	45.8 - 51.3	
0 to 8 years	16,414	70.4	69.2 -71.5	53.6	52.3 - 54.9	
Social Class						
A	240	1.5	1.1 - 2.0	39.8	30.0 - 50.6	0.0023
B	2,810	13.8	12.9 - 14.7	47.8	44.9 - 50.8	
C. D. and E	19,675	84.7	83.7 - 85.7	52.6	51.4 - 53.7	
Residential Area						
Urban	17,313	85.5	84.3 - 86.1	52.5	51.2 - 53.7	<0.0001
Rural	5,415	14.5	14.0 - 15.2	46.3	43.7 - 48.9	
Region of Residence						
Southeast	5,825	47.3	46.3 - 48.4	52.8	50.8 - 54.7	<0.0001
South	3,307	16.2	15.5 - 16.9	54.1	51.8 - 56.5	
Central-West	2,373	6.26	5.9 - 6.62	52.3	49.5 - 55.1	
North	3,487	5.56	5.3 - 5.9	43.7	40.9 - 46.5	
Northeast	7,736	24.7	23.9 - 25.5	49.4	47.4 - 51.3	

Source: National Health Survey, PNS, 2019.

Note:

a Population proportion;

b 95% Confidence Interval;

c Probability value for the Chi-square test. ^^*^^ Self-reported yellow or indigenous skin color, with two missing values.

*Source: National Health Survey, PNS, 2019.*

**Figure 1:**
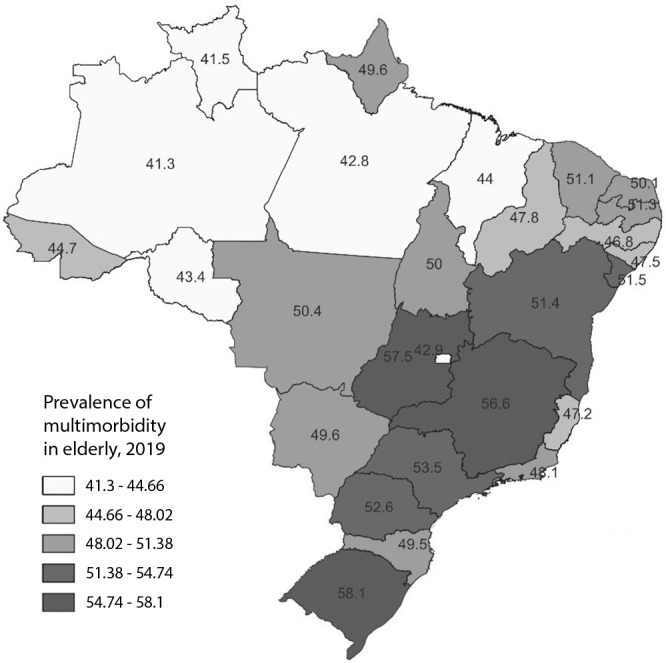
Prevalence of multimorbidity in elderly individuals according to Brazilian States. Brazil, 2019

Statistically significant differences were observed in the prevalence of multimorbidity among Brazilian states (p<0.0001). The highest prevalences were found in the South, Central-West, and Southeast regions, specifically in Rio Grande do Sul (58.1%; 95% CI: 54.2-61.9), Goiás (57.5%; 95% CI: 53.0-61.9), and Minas Gerais (56.6%; 95% CI: 52.6-60.4), respectively. The lowest prevalences of two or more diseases in the elderly were observed in the North and Northeast regions, with Sergipe in the Northeast having the highest prevalence of multimorbidity (51.5%; 95% CI: 46.2-56.6) (Figure 1).

In the unadjusted association analysis, it was found that being female (unadjusted PR = 1.36; 95% CI: 1.28-1.40); being 80 years or older (unadjusted PR = 1.19; 95% CI: 1.10-1.27); having low education, with 0 to 8 years of study (unadjusted PR = 1.19; 95% CI: 1.10-1.28); belonging to a low social class - C, D, or E (unadjusted PR = 1.32; 95% CI: 1.01-1.71); having smoked in the past (unadjusted PR = 1.10; 95% CI: 1.06-1.15); engaging in insufficient physical activity (unadjusted PR = 1.18; 95% CI: 1.11-1.16); and spending three hours or more in front of screens (unadjusted PR = 1.16; 95% CI: 1.12-1.21) were factors associated with an increase in the prevalence of multimorbidity in elderly individuals. Meanwhile, living in the North and Northeast regions, as well as in rural areas, were associated with lower prevalences of multimorbidity (Table 2).

**Table 2:** Unadjusted and adjusted analyses of the associations between sociodemographic characteristics, lifestyle, and anthropometry with the occurrence of multimorbidity in elderly individuals. Brazilian States, Brazil, 2019

Variables	Multimorbidity			
	Unadjusted PR^a^ (95% CI)	*p* value	Adjusted PR^b^ (95% CI)	*p* value
Gender (reference: male)				
Female	1.36 (1.28-1.40)	<0.001	1.33 (1.27-1.39)	<0.001
Age Group (reference: 60-69)				
70-79	1.15 (1.10-1.20)	<0.001	1.11 (1.06-1.16)	<0.001
80 or more	1.19 (1.10-1.27)	<0.001	1.12 (1.05-1.19)	<0.001
Education (reference: 12 or more)				
9 - 11	1.08 (0.98-1.19)	0.094	1.06 (0.97-1.17)	0.156
0 - 8	1.19 (1.10-1.28)	<0.001	1.16 (1.07-1.25)	<0.001
Social Class (reference: A)				
B	1.20 (0.91-1.57)	0.183	-	-
C, D and E	1.32 (1.01-1.71)	0.038	-	-
Residential Area (reference: urban)				
Rural	0.88 (0.83-0.93)	<0.001	0.91 (0.85-0.96)	0.003
Region of Residence (reference: Southeast)				
South	1.02 (0.96-1.08)	0.391	1.04 (0.98-1.10)	0.150
Central-West	0.99 (0.92-1.05)	0.766	1.01 (0.95-1.08)	0.629
North	0.82 (0.76-0.89)	<0.001	0.83 (0.78-0.90)	<0.001
Northeast	0.93 (0.88-0.98)	0.015	0.93 (0.88-0.98)	0.012
Smoking (reference: Never smoked)				
Currently smokes	0.89 (0.82-0.96)	0.006	0.93 (0.86-1.01)	0.119
Smoked in the past	1.10 (1.06-1.15)	<0.001	1.16 (1.11-1.21)	<0.001
Alcohol Consumption (reference: Never consumes)				
Light/Moderate consumption	0.85(0.71-1.03)	0.104	-	-
Heavy consumption	1.11 (0.94-1.31)	0.211	-	-
Physical Activity Practice (reference: Sufficient)				
Insufficient	1.18 (1.11-1.26)	<0.001	1.13 (1.06-1.21)	<0.001
Body Mass Index (reference: Normal weight)				
Underweight	1.13 (0.90-1.42)	0.270	-	-
Overweight/Obesity	1.12 (0.98-1.27)	0.076	-	-
Screen Time (reference: Less than 3 hours/day)				
More than 3 hours/day	1.16 (1.12-1.21)	<0.001	1.13 (1.08-1.18)	<0.001

Source: National Health Survey, 2019.

Note: Ref: reference;

a Unadjusted PR: Unadjusted Prevalence Ratio;

b Adjusted PR: Prevalence Ratio adjusted for all independent variables in the multiple model; 95% CI: 95% Confidence Interval.

In the multivariate analysis, multimorbidity remained associated with sex (adjusted PR = 1.33; 95% CI: 1.27-1.39), the age group of 70-79 years (adjusted PR = 1.11; 95% CI: 1.06-1.16), and being 80 years or older (adjusted PR = 1.12; 95% CI: 1.05-1.19), showing a dose-response effect with increasing age (p for trend <0.001), and having low education - 0 to 8 years of study (adjusted PR = 1.16; 95% CI: 1.07-1.25). Residing in the North (adjusted PR = 0.83; 95% CI: 0.78-0.90) and Northeast (adjusted PR = 0.93; 95% CI: 0.88-0.98) regions appeared protective against multimorbidity in elderly individuals, compared to living in the Southeast. Additionally, residing in a rural area was negatively associated with the presence of two or more chronic diseases (adjusted PR = 0.91; 95% CI: 0.85-0.96).

Regarding lifestyle factors, previous tobacco use (adjusted PR = 1.16; 95% CI: 1.11-1.21), insufficient physical activity (adjusted PR = 1.13; 95% CI: 1.06-1.21), and screen time - three or more hours per day (adjusted PR = 1.13; 95% CI: 1.08-1.18) remained positively associated with multimorbidity in the multivariate model (Table 2).

## DISCUSSION

In this research, it was observed that more than half of the elderly population residing in households in Brazil exhibited multimorbidity. This finding aligns with other national studies, which reported a similar proportion of 57.1%^(18)^. These estimates are close to those observed in high-income countries, where prevalence can reach 66.1%, indicating a high burden of this condition in the elderly population in low, middle, and high-income countries^(19)^. This result has implications for health promotion policies and for health professionals, especially those working in primary care, to facilitate improvements related to health promotion and education, particularly in younger age groups, aiming to reduce the impact of the coexistence of two or more diseases in the same individual over time.

It is highlighted that elderly Brazilian women may have a higher probability of having two or more chronic conditions, a reality also observed in international contexts^(19-20)^. Their longer life expectancy prolongs their time of exposure to risk factors for chronic conditions, leading to a greater propensity for multimorbidity^(21-22)^. This is in addition to the decline in estrogen production, a hormone directly linked to cardiovascular and bone protective factors^(23)^. Gender-related factors also influence, as women tend to seek more health services due to aspects of sexuality and reproduction^(24)^.

Elderly individuals in higher age groups may be more prone to this outcome due to the decline of their physiological functions and longer exposure to situations that can trigger chronic diseases. This dose-response effect of age in relation to multimorbidity was also observed in previous research in high-income countries^(19-20)^.

Social determinants, especially low education, can exacerbate the occurrence of the simultaneity of two or more chronic conditions in the same individual. Low education is directly related to socioeconomic level and can impact access to health services and the promotion of self-care, making it difficult to understand a healthy lifestyle and justifying the higher chance of multimorbidity and poorer quality of life^(25)^.

Therefore, health professionals must be aware that a large portion of the population with multimorbidity has low education. This characteristic affects understanding of the health-disease process, food choices, adherence to prevention and treatment measures, and difficulty in accessing health care. It is necessary to expand the connection with these users, as this dimension of care in primary attention is associated with positive outcomes in the user’s trust in the professional, more precise treatments, and knowledge about the patient’s preferences, values, and social context, which are paramount for care tailored to individual needs, considering the social determinants of health^(26)^.

It is notable that there are significant differences in the prevalences of multimorbidity among elderly individuals in different regions and residential areas in Brazil. The Northeast and North regions show lower prevalences of multimorbidity compared to the Southeast and South. This result is consistent with other research of national representativeness^(18)^. This finding could be related to the lifestyle adopted by elderly individuals in these regions^(27)^, as well as to the higher life expectancy in the South of the country, increasing the period of exposures and susceptibility to the occurrence of two or more chronic health conditions, thus justifying the higher prevalences of the outcome observed in the Southern region^(28)^.

Differences in the prevalences of multimorbidity are also observed between urban and rural areas in Brazil. Residing in rural areas may decrease the prevalence of multimorbidity. However, this association could be related to underreporting in the diagnosis of chronic health conditions in rural areas, due to difficulties in accessing health services in these areas. International studies point to greater vulnerability of rural populations regarding the health service offered^(29)^. Nevertheless, the prevalences of multimorbidity in international studies with elderly individuals residing in rural areas were similar to those identified in this research, reaching up to 48.8% in rural India^(14)^.

Behavioral factors, such as past smoking habits, have been associated with multimorbidity. Smoking is a known risk factor for various non-communicable chronic diseases (NCDs). Exposure to toxins, like nicotine, increases the incidence of cancer, chronic respiratory diseases, and others, in addition to multimorbidity^(30)^. Research has observed the potential role of smoking and the consumption of tobacco products, along with excessive alcohol consumption, in the occurrence of multimorbidity^(27)^.

Insufficient physical activity in elderly individuals can increase the prevalence of two or more associated chronic diseases. There is strong evidence that insufficient physical activity is associated with a variety of chronic conditions and multimorbidity^(31-32)^. The World Health Organization (WHO) recommendations for people aged 65 or older include at least 150 minutes of moderate-intensity physical exercise or at least 75 minutes of intense or vigorous activity per week^(14)^.

Practicing activities as recommended can reduce the risk of developing cardiovascular diseases and their metabolic risk factors, bringing benefits for immunity, respiratory capacity, as well as improving the social and psychological aspects of individuals^(33)^. In this context, programs to encourage the practice of physical activity for leisure in elderly people, such as the Health Academy Program (PAS), should be promoted and enhanced as a Primary Health Care strategy. Additionally, at this level of care, health professionals should encourage the formation of community groups with incentives for group activities among elderly people, aiming to mitigate physical inactivity, which has a potential impact on the development of NCDs and multimorbidity.

Sedentary behavior, measured by screen use for 3 or more hours a day, can be associated with higher prevalences of two or more chronic health conditions. Research shows that the lack of locations for physical activity near residences can increase the likelihood of sedentary behavior in elderly people^(34)^.

In this sense, public health promotion policies for the elderly should consider the use of locations near residences that encourage the practice of physical activities. Health professionals should encourage elderly individuals to interrupt long periods of sitting, alternating them with standing positions, as an alternative to reduce the impact of spending a lot of time in activities with low caloric expenditure, which are associated with type II diabetes, cancer, cardiovascular diseases, obesity, multimorbidity, and mortality from all causes^(34-36)^.

### Study Limitations

This study has some limitations, related to the possibility of reverse causality due to the cross-sectional nature of the data. The relationships found are limited to associations and do not imply causality. Additionally, other factors from the social and physical environments where individuals live were not considered. On the other hand, an asymptotic sample distributed across all Brazilian states was used, which provides external validation and estimates close to population parameters. Most of the estimates presented in this research are consistent with the results of studies conducted in high and middle-income countries.

### Contributions to Nursing, Health, and Public Policy

The findings of this study offer insights into the magnitude of multimorbidity in the elderly population, facilitating the assessment of current public policies for the prevention and control of NCDs. Moreover, the results can contribute to the development of public health interventions and nursing practices, regarding the promotion of clinical guidelines and health education, focusing on stimulating changes in modifiable factors related to the occurrence of multimorbidity.

The role of nursing in providing guidance about the harms of screen use for 3 or more continuous hours a day, the adverse effects of smoking on the onset of chronic diseases and multimorbidity, and the encouragement of regular physical activity with appropriate frequency and intensity is highlighted.

Furthermore, the focus of public health policies, social interventions, and the actions of health and nursing professionals should primarily be on women and people with low education, who were identified as the most vulnerable subgroups to multimorbidity. It is emphasized that health and nursing interventions should be transversal and interdisciplinary, covering not only the elderly but all life cycles, considering that aging is a continuous and heterogeneous phenomenon in which nursing plays a significant role in promoting healthy aging.

## CONCLUSIONS

Using representative data from Brazil, our results revealed that more than half of the elderly in the country have two or more chronic health conditions. This finding underscores multimorbidity as a significant public health problem, given its high frequency in this population. Significant differences in the occurrence of multimorbidity among states, regions, and rural and urban areas in Brazil were observed, with the Southern region presenting the highest burden of this outcome among the elderly.

Multimorbidity was associated with social, demographic, and behavioral factors, including female sex, older age groups (70 years or more), low education (zero to eight years of study), previous smoking, region and area of residence, insufficient physical activity, and screen use for three or more hours daily.

The role of health professionals, especially those working in primary care, in strengthening their bond with these individuals is emphasized, aiming to provide individualized care attentive to the social determinants of health. Understanding multimorbidity as a complex challenge requires intersectoral efforts and the coordination of health promotion, disease prevention, and control actions, encompassing not only the elderly but also children, adolescents, and adults, recognizing aging as a complex and continuous phenomenon.
